# Bioactivities of rose-scented geranium nanoemulsions against the larvae of *Anopheles stephensi* and their gut bacteria

**DOI:** 10.1371/journal.pone.0246470

**Published:** 2021-02-08

**Authors:** Maryam Dehghankar, Naseh Maleki-Ravasan, Azar Tahghighi, Fateh Karimian, Mohsen Karami

**Affiliations:** 1 Faculty of Basic Science, Science and Research Branch, Islamic Azad University, Tehran, Iran; 2 Malaria and Vector Research Group, Biotechnology Research Center, Pasteur Institute of Iran, Tehran, Iran; 3 Department of Parasitology, Pasteur Institute of Iran, Tehran, Iran; 4 Laboratory of Medicinal Chemistry, Department of Clinical Research, Pasteur Institute of Iran, Tehran, Iran; 5 Department of Medical Entomology and Vector Control, School of Public Health, Tehran University of Medical Sciences (TUMS), Tehran, Iran; 6 Department of Parasitology and Mycology, Babol University of Medical Sciences, Babol, Iran; Al-Azhar University, EGYPT

## Abstract

*Anopheles stephensi* with three different biotypes is a major vector of malaria in Asia. It breeds in a wide range of habitats. Therefore, safer and more sustainable methods are needed to control its immature stages rather than chemical pesticides. The larvicidal and antibacterial properties of the *Pelargonium roseum* essential oil (PREO) formulations were investigated against mysorensis and intermediate forms of *An*. *stephensi* in laboratory conditions. A series of nanoemulsions containing different amounts of PREO, equivalent to the calculated LC_50_ values for each *An*. *stephensi* form, and various quantities of surfactants and co-surfactants were developed. The physical and morphological properties of the most lethal formulations were also determined. PREO and its major components, i.e. citronellol (21.34%), L-menthone (6.41%), linalool (4.214%), and geraniol (2.19%), showed potent larvicidal activity against the studied mosquitoes. The LC_50/90_ values for mysorensis and intermediate forms were computed as 11.44/42.42 ppm and 12.55/47.69 ppm, respectively. The F48/F44 nanoformulations with 94% and 88% lethality for the mysorensis and intermediate forms were designated as optimized formulations. The droplet size, polydispersity index, and zeta-potential for F48/F44 were determined as 172.8/90.95 nm, 0.123/0.183, and -1.08/-2.08 mV, respectively. These results were also confirmed by TEM analysis. Prepared formulations displayed antibacterial activity against larval gut bacteria in the following order of decreasing inhibitory: LC_90_, optimized nanoemulsions, and LC_50_. PREO-based formulations were more effective against mysorensis than intermediate. Compared to the crude PREO, the overall larvicidal activity of all nanoformulations boosted by 20% and the optimized formulations by 50%. The sensitivity of insect gut bacteria may be a crucial factor in determining the outcome of the effect of toxins on target insects. The formulations designed in the present study may be a good option as a potent and selective larvicide for *An*. *stephensi*.

## Introduction

Mosquitoes (Diptera: Culicidae) are considered as the deadliest creatures in the world since they carry and spread various diseases such as malaria, dengue, West Nile fever, Encephalitis, Rift Valley fever, yellow fever, Zika, chikungunya, and lymphatic filariasis to humans, resulting in millions of death annually [1]. In 2018, malaria alone caused the death of 405,000 people globally, compared with 416,000 estimated death in 2017 and 585,000 in 2010 [2].

A number of ~40 species of *Anopheles* are recognized as the dominant vectors of malaria [3]. Among these species, *An*. *stephensi*, the Asian malaria mosquito, is widely distributed from the Middle East to the Indian subcontinent and Southeastern Asia [4–8]. Based on the egg phenotypes, *An*. *stephensi* has three biological forms (BFs), which type form is known to be anthropophilic and a more competent vector of urban malaria, whereas mysorensis and intermediate forms are regarded as relatively zoophilic and poor vectors in the rural areas [9]. All three forms happen in diverse human environments in Iran. In Hormozgan Province, the type and intermediate forms of *An*. *stephensi* are found in urban coastal or suburban/rural plain regions, whereas the mysorensis is observed only in rural mountainous localities. Conversely, in Sistan and Baluchestan Province, only mysorensis form has been reported from different settings and climates [10, 11]. *An*. *stephensi* is recognized as a proficient vector for both *Plasmodium falciparum* and *P*. *vivax*, the agents of clinically severe malaria [9], and also for *Plasmodium* species, the causative agents of rodent malaria [12].

Larvae of *An*. *stephensi* are found in a wide variety of breeding habitats such as fresh and brackish waters in rural, coastal and urban areas. In rural areas, the larvae exploit freshwater pools, stream banks and bottoms, catch basins, seepage canals, wells, and local water storage containers. In urban areas, they readily breed in numerous artificial containers inside and outside homes, as well as in industrial regions [9, 13, 14].

Targeting mosquito vectors to interrupt the circulation of pathogens has always been a basic control strategy against major mosquito-borne diseases [15]. Early mosquito control policies were principally relied on the larval source management, through larviciding and biological control agents, together with environmental modifications [16, 17]. In the early 1940s, the chemical era of vector control was launched with organochlorine DDT usage, both as larvicide and adulticide [18]. Over the time, two groups of organophosphates and carbamates, potent cholinesterase inhibitors, were largely replaced by organochlorines [19, 20]. In the 1980s, synthetic pyrethroids compounds were added to the arsenal of public health insecticides [21]. As modern insecticides, these compounds are broadly recommended for in-home insect control, as well as for the treatment of mosquito nets and other materials [22, 23].

Despite the merits of chemical pesticides in vector control, there are still issues undermined the achievements of eliminating or controlling major mosquito-borne diseases. Chemicals may cause both acute and delayed health effects in exposed individuals [24]. In addition, the nonstop use of pesticides gives a rise to the environmental pollution and disruption of natural and biological control systems [25–27]. The emergence and spread of resistance to pesticides are highly serious issues, which in turn have increased the dosage of pesticides and the quest for stronger and safer alternatives [28]. Approaches that may help reduce reliance on synthetic pesticides are mainly comprised of biological control [29], transgenic and paratransgenic methods [30–33], as well as plant essential oils (EOs) that can act as green pesticides [34–36].

EOs are a blend of naturally occurring volatile aromatic and aliphatic compounds manufactured in plants as secondary metabolites [37, 38]. These oils are important in biosciences for their antibacterial, antiviral, antifungal, antiparasitic, anticancer, insecticidal, psychophysiological, neuroprotective and anti-aging activities [38]. The reason why EOs are effective against a broad variety of pathogens is attributed to the existence of different chemical families of alcohols, ethers or oxides, aldehydes, ketones, esters, amines, amides, phenols, heterocycles, and chiefly the terpenes [39]. In recent years, nanoemulsion-based delivery systems have been the focus of many studies. Thanks to their subcellular size, nanocarriers have potential to boost the bioactivity of EOs since they allow a wide tissue penetration and an effortless cellular uptake. Additionally, they render possible to adjust the release of active ingredients at the target site. The nanoemulsion-based delivery systems have been proposed to ameliorate the EOs’ physico-chemical properties by decreasing their volatility, improving the stability, and enhancing water solubility, as well as by protecting them from the interaction with the environment [40, 41]. These formulations may correspondingly enhance other characteristics, namely phytotoxicity (when applied on vegetation [42]) and spraying improvements (i.e. fog treatments [43]). Nanoemulsion of different plant EOs has hitherto been prepared, and their mosquito larvicidal properties have been investigated [44–53].

The idea that insects are colonized by numerous microorganisms, particularly bacteria, has widely been acknowledged by life scientists. The external and internal parts of the insect body, especially digestive tract, offer conditions and resources needed to support beneficial microbiota [54]. Several bacterial symbionts promote host fitness by contributing to nutrition [55], reproduction [56, 57], speciation [58], immunity/defense [59–62], ecological communication [63], and pathogen transmission ability [64]. More recent studies have pinpointed that there is a potential linkage between insect gut microbiota and their susceptibility to insecticides [65–67]; therefore, the sensitivity of gut bacteria needs to be taken into account prior to any intervention measures.

In spite of its supreme biological potential, the *Pelargonium roseum* essential oil (PREO) remains, to our knowledge, unexplored concerning the development of an innovative nanolarvicide. Therefore, the present study aimed to assess the larvicidal activity of PREO and its components, to prepare various nanoemulsions of PREO, to select optimized formulations in terms of physical and biological properties and to evaluate the interaction of larval gut bacteria with PREO formulations as a mechanism for the attribution of the oil insecticidal activity.

## Materials and methods

### Chemical materials

*Pelargonium roseum* (Geraniaceae) essential oil (PREO; Geranium; batch no. 92/2) obtained from Barij Essence Pharmaceutical Co. (Iran) was kept in a refrigerator at 4°C away from direct sunlight. Tween 20 (TW), ethanol (ETH), and chemical constituents of PREO (citronellol [cat no. 27470], geraniol [cat no. 163333], linalool [cat no. L2602], L-menthone [cat no. W266701]) were acquired from Sigma-Aldrich Company (Germany). Technical grade of temephos (97.3%, Pestanal ®; Sigma-Aldrich, Riedel-de Haën, Germany) was received from Institute Pasteur du Laos. The bacterial propagation media, such as brain heart infusion (BHI) broth (M210) and BHI agar (M211), were procured from HiMedia Laboratories, Mumbai, India. Volumetric instruments of class A with the highest grade accuracy were used.

### Biological materials

Late third and fourth instars larvae of two BFs of *An*. *stephensi* (mysorensis and intermediate) were purchased from the National Insectarium of Iran, Malaria and Vector Research Group. These strains were maintained separately in the laboratory conditions according to the MR4 standard protocols [68]. The origins of *An*. *stephensi mysorensis* and *An*. *stephensi intermediate* forms were from Chabahar City in Sistan and Baluchestan Province in Southeastern Iran and from Bandar Abbas City in Hormozgan Province in Southern Iran, respectively. Prior to any experiment, the BFs of the *An*. *stephensi* specimens were determined by counting the number of egg ridges, as described in the literature [69].

### Identification of the PREO constituents

Chemical constituents of PREO were characterized by gas chromatography-mass spectrometry (GC-MS), which was achieved on a GC (HP 6890, Agilent, USA) equipped with a mass spectrometer detector (HP 5973, Agilent). The MS was operated in the electron ionization mode (70 eV). The MS ion source temperature and the MS quadrupole temperature were kept at 230°C and 150°C, respectively. Afterwards, 1 μL of the diluted sample (10 μL in 1 mL of heptane) was injected by autosampler using a 100:1 split ratio and analyzed on a capillary column (TRB-5MS, 30 m, 250 μm, and 0.25 μm). Helium functioned as the carrier gas (99.9995% pure), and its flow rate in the column was adjusted to 1 mL/min^-1^. The sample was assessed under the following settings: initial oven temperature at 36°C for 5 min, ramp-up at 4°C/min to 200°C and continued for 8 min, then increased up to 280°C with a ramp-up of 40°C/min for 10 min and overall run time of 66 min.

Compounds were determined by comparing their respective mass spectra, retention time, and relative abundance of acceptance match criteria with those of standards and also by comparing with the NIST05 (National Institute of Standards and Technology, Gaithersburg, MD, USA) and the Wiley Registry of Mass Spectral Libraries. The retention were indices calculated for each component using a mixture of n-alkanes (C9-C24) dissolved in n-hexane based on the following formula: Retention Indices = 100 × [n + (Tu-Tn)/(TN-Tn)]

Where n = the number of carbons in the alkane preceding compound; N = the number of carbons in the alkane following compound; Tu = the retention time of the unknown compound; Tn = the retention time of the preceding alkane; TN = the retention time of the following alkane.

### Larvicidal activity of PREO and its main constituents

Larvae of the two BFs of *An*. *stephensi* were exposed to seven (1.56 to 100 ppm) serially diluted concentrations of the PREO for 24 h, according to the methods described previously [35, 70, 71]. Due to the hydrophobic behavior of PREO, the oil was initially dissolved in ETH 96% as stock (100 ppm = 100 μg/ml), and subsequent concentrations were prepared by the stock dilution. Also, larvicidal activities of the main components of the PREO (citronellol, L-menthone, linalool, and geraniol) were evaluated both individually and in combination at two concentrations equal to LC_50/90_ values in the same way. The components were evaluated individually based on the LC_50_ (11.44 and 12.55 μl) and LC_90_ (42.42 and 47.69 μl) values for the Chabahar and Bandar Abbas strains, respectively; the volume of each constituent increased up to 1000 μl with ETH. In the combined mode, each component under study had a concentration equivalent to a chemical composition in the crude PREO. Thus, for Chabahar strain, each component of citronellol, L-menthone, linalool, and geraniol at concentrations equivalent to LC_50_ (11.44 ppm containing 7.15, 2.14, 1.41, and 0.74 μL, respectively) and LC_90_ (42.42 ppm containing 26.51, 7.96, 5.23, and 2.72 μL, respectively) were prepared and made to a volume of 1 ml with ETH. For Bandar Abbas strain, the solutions were also made based on LC_50_ (12.55 ppm containing 7.84, 2.36, 1.55, and 0.80 μL, respectively) and LC_90_ (47.69 ppm containing 29.80, 8.95, 5.88, and 3.06 μL, respectively) values.

For all treatments, 1 mL of each concentration of the PREO solution was supplemented with 99 mL of the dechlorinated water containing 0.001% TW 20, to make up 100 mL of test solution in a 200-mL glass beaker. A plastic rod was applied to stir the oil-ethanol-water solution for 30 s. Four different beakers, one containing 0.125 mg/L of temephos (13.2 μL in 1mL ETH), one comprising of 1% ETH, one holding of 0.001% TW, and the other including untreated dechlorinated water, were set as control solutions. Batches of ~20 healthy larvae were gently transferred to the beakers by a fine strainer. The bioassay was carried out in a room with the conditions of 24 ± 1°C, 50 ± 5% relative humidity, and 12:12 light and dark photoperiodicity. After 24 h of exposure, larval mortality was observed, and four replicates was used for Probit analysis and LC_50/90_ calculation. If the mortality in the untreated group was between 5% and 20%, the bioassay was then corrected via Abbott’s formula [72].

### Preparation of PREO nanoemulsions

To reduce the volatility and improve aqueous solubility of PREO, 16 oil-in-water (O/W) nanoemulsions of the oil were prepared, as described previously [50]. Briefly, different concentrations of TW 20 (2–16%) and ETH 96% (2–32%) were mixed at 600 rpm at room temperature for 5 minutes. Amounts of 11.44 and 12.55 ppm of PREO, equivalent to the calculated percentage of LC_50_ values for *An*. *stephensi mysorensis* and *An*. *stephensi intermediate*, respectively, were added to the TW (as surfactant) and ETH (as co-surfactant) mixture and stirred at the same conditions for 15 min. Subsequently, deionized water was gradually supplemented up to 10 mL and stirred further for 30 min.

### Characterization of PREO nanoemulsions

The larvicidal activity of 16 PREO nanoemulsions (F22-F1632) was investigated against BFs of *An*. *stephensi*, and the physical properties of the most lethal ones were determined. The oil particle size (PS), polydispersity index (PDI), and zeta-potential (ZP) were measured by dynamic light scattering (DLS) with a Zetasizer Nano ZS (Malvern, ZEN3600, UK) working at 633 nm at 25°C and equipped with a backscatter detector at 173°. The droplets’ morphology of optimized formulations was also determined by transition electron microscopy (TEM) (RASTAK Lab; Tehran, Iran). The appearance and ZP of the optimum nanoemulsions stored at 4°C were checked after 40 days.

### Comparison of the larvicidal activity of optimized nanoformulations with PREO

Larvicidal activities of optimized nanoemulsions, F48 for *An*. *stephensi mysorensis* and F44 for *An*. *stephensi intermediate*, were precisely performed as mentioned before. The mortality caused by each formulation was compared with their corresponding PREO LC_50_, 11.5 ppm for *An*. *stephensi mysorensis* and 12.5 ppm for *An*. *stephensi intermediate*.

### Interaction of larval aerobic gut bacteria with PREO and optimized nanoemulsions

Five fourth instar larvae of each *An*. *stephensi* form were randomly selected for dissection and identification of gut-associated bacteria. Before dissection, specimens were washed twice thoroughly with sterile PBS (phosphate buffer saline) 1× and then surface sterilized in 70% ETH for 2 min. Guts were removed aseptically within a sterile PBS drop on a microscopic slide under a microbiological laboratory hood. Then guts were mechanically grinded in 1.5-ml Eppendorf tubes containing 100 μl of PBS. The homogenates were inoculated into BHI broth medium and subsequently plated on the BHI agar medium to obtain pure colonies. Colonies with different phenotypes were isolated and sub-cultured successively. Individual bacteria were identified using *16S rDNA* sequencing method, as described in the experiments [31]. Based on the frequency of colony-forming units (CFU) on the BHI agar medium, two bacterial isolates, one Gram-positive bacterium and one Gram-negative bacterium, were chosen from each form of *An*. *stephensi* to explore the interaction of bacteria with the PREO formulations.

In each interaction test, 32.650 ml of BHI broth, 330 μl of each PREO dilution (equal volume of the LC_50/90_ for each biological form as well as their corresponding optimized nanoemulsions), and 20 μl of suspension of each target microorganism (adjusted to 0.5 McFarland standard turbidity corresponding to 10^8^ CFU/mL) were mixed together. The interacting mixtures were incubated at 37°C and shaken at 200 rpm for 24 h. The growth of bacteria was quantified by measuring the OD at 600 nm (Eppendorf BioPhotometer plus, Germany) at different time points, i.e. 2, 4, 8, 12, and 24 hours post interaction (hpi). Experiments were performed in triplicate. Four culture media, comprising of one medium without any additives and two media containing TW and ETH (as negative controls), and a medium including only the desired bacterium (as positive control), were used for the clarification of the interaction results. The percentage inhibition of the growth of bacteria exposed to treatments during 24 h was calculated by the formula: % inhibition = |Test OD—Control OD/Control OD| × 100, where the test group OD corresponded to bacteria treated with antimicrobial compound concentrations, and the control group corresponded to the untreated bacteria grown under normal conditions.

### Statistical analysis

The design was a comparative study on the efficacy of the various formulations of PREO in the mortality of the larvae of two BFs of *An*. *stephensi*. The LC_50_ and LC_90_ values for the respective BFs were calculated using probit analysis according to the method described previously [71]. The LC_50_ and LC_90_ values were chosen based on chi-squared values and degrees of freedom. The significance of the slope probit-log (dose) regression was assessed using the z test (z = $\frac{\beta }{\sigma (\beta )}$), if the *P* values less than 0.05 represented correlations between PREO doses and mortalities. The heterogeneity factor *h* of the regression equation was calculated to adjust for large χ2. *h* was defined as h = $\frac{\chi 2}{df}$ If *h* < 1, the model provided a good fit to the data [73]. SPSS V22.0 software was used for data analysis. Graphs were drawn using GraphPad Prism® v.5.00 (GraphPad Software Inc, San Diego, CA, USA). Mean ± standard deviation (SD) was used to describe quantitative variables. Percentage and frequency were also applied for qualitative variables. The assumptions of normality and homogeneity of variance were tested by Shapiro-Wilks and Brown-Forsythe tests, respectively. The data did not violate these assumptions. The two-tailed Student’s t test was used to comparison of larvicidal activity of optimized nanoformulations with corresponding PREO LC_50_. The One-way ANOVA analysis was used to compare the larval mortalities of two BFs exposed to components of PREO, as well as to evaluate growth inhibition of gut bacteria exposed to diverse PREO formulations. If ANOVA test was significant, Tukey’s post-hoc analysis was applied. Significance level was considered less than 0.05.

## Results

### BFs of the *An*. *stephensi*

The BFs of *An*. *stephensi* strains of Chabahar and Bandar Abbas were confirmed as mysorensis and intermediate, respectively, based on egg ridges counts (detailed results are not shown).

### Chemical composition of PREO

The GC-MS analysis revealed the presence of 36 constituents in the PREO, of which only 19 volatile compounds with similarity ≥90% were detected using available libraries, corresponding to 81.79% of the total oil. The four major components, citronellol (21.34%), L-menthone (6.41%), linalool (4.214%), and geraniol (2.19%), identified with the similarity of ≥96% were subjected to larvicidal bioassay (Table 1).

**Table 1:** Inventory of components identified in *Pelargonium roseum* essential oil using GC-MS analysis.

Compounds	Molecular weight (g/mol)	Formula	Retention indices*	Percentage of area (% identity)
α-Pinene	136.23	C_10_H_16_	930	1.82(97)
β-Pinene	136.23	C_10_H_16_	977	0.65(94)
Ocimene	136.23	C_10_H_16_	1045	0.912(93)
Ocimene quintoxide	154.25	C_10_H_18_O	1056	0.92(95)
Linalool oxide	170.25	C_10_H_18_O_2_	1074	4.49(91)
Linalool	154.25	C_10_H_18_O	1101	4.22(97)
6-Methyl-3,5-heptadien-2-one	124.18	C_8_H_12_O	1104	0.64(90)
cis-Rose oxide	154.25	C_10_H_18_O	1109	3.14(96)
trans-Rose oxide	154.25	C_10_H_18_O	1128	1.55(93)
trans-Ocimenol	152.23	C_10_H_16_O	1134	2.93(96)
L-Menthone	154.25	C_10_H_18_O	1166	6.41(98)
α-Terpineol	154.25	C_10_H_18_O	1197	3.56(94)
Citronellol	156.26	C_10_H_20_O	1246	21.34(98)
Geraniol	154.25	C_10_H_18_O	1264	2.19(96)
Citronellyl formate	184.27	C_11_H_20_O_2_	1282	12.64(91)
Geranyl formate	182.26	C_11_H_18_O_2_	1304	1.46(92)
Citronellyl acetate	198.3	C_12_H_22_O_2_	1356	9.82(93)
β-Bourbonene	204.35	C_15_H_24_	1375	1.89(96)
cis-Calamenene	202.33	C_15_H_22_	1510	1.21(90)

*The retention indices were calculated for each component using a mixture of n-alkanes (C9-C24) dissolved in n-hexane.

### Larvicidal activity of PREO and its main constituents

**Fig 1:**
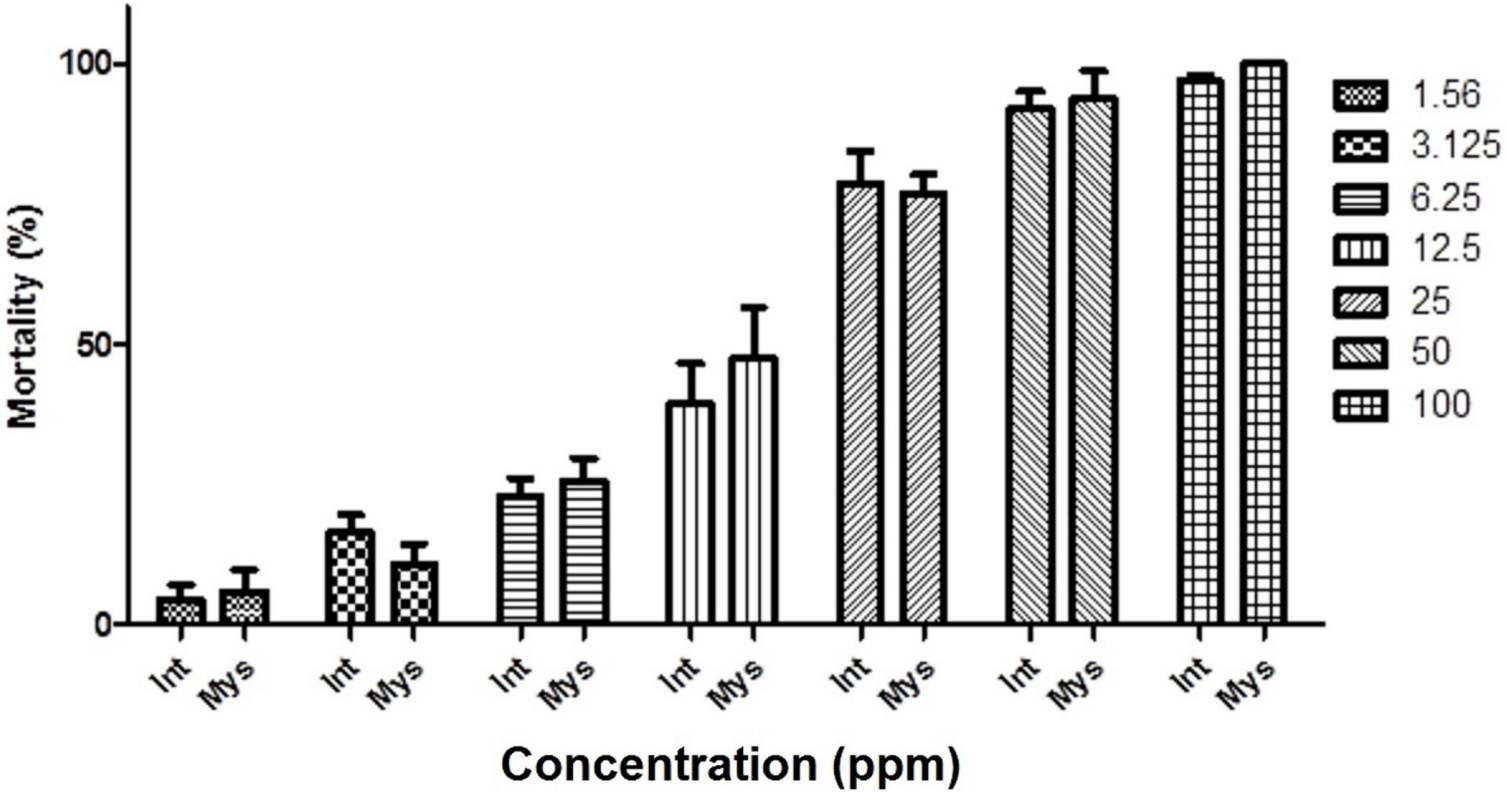
Larvicidal activity of *Pelargonium roseum* essential oil against mysorensis and intermediate forms of *Anopheles stephensi*.

After 24 h exposure, the PREO showed potent larvicidal activity against BFs of *An*. *stephensi*. The mortality in the control groups did not exceed 5% in all concentrations; thus, there was no need for corrections. Temephos caused ~90% mortality in both mysorensis and intermediate forms. Mortality of larvae exposed to PREO increased in a dose-dependent manner (Table 2). Minimum and maximum larval mortality for both BFs were determined to be at 1.56 ppm and 100 ppm concentrations, respectively (Fig 1). The LC_50_ and LC_90_ values against the larvae of *An*. *stephensi mysorensis* were 11.44 and 42.42 ppm, while they were 12.55 and 47.69 ppm for *An*. *stephensi intermediate*, respectively (Table 2).

**Table 2:** Probit regression line parameters of two biological forms of *Anopheles stephensi* to *Pelargonium roseum* essential oils at different interval concentrations.

Biological forms	B ± SE	LC_50_ (LCL- UCL) 95% C.I.(ppm)	LC_90_ (LCL-UCL) 95% C.I.(ppm)	χ2(df)	H^a^	*P* value^b^
*An*. *stephensi mysorensis* (Chabahar)	2.25 ± 0.31	11.44	42.42	1.31(5), NS	0.26	< 0.05
		(8.52–15.36)	(29.00–76.48)			
*An*. *stephensi intermediate* (Bandar Abbas)	2.21 ± 0.28	12.55	47.69	3.43(5), NS	0.68	< 0.05
		(9.584–16.53)	(33.16–82.16)			

B, slope; SE, standard error; LC_50_, 95% CI, lethal concentration causing 50% mortality and its 95% confidence interval; L/UCL, lower/upper confidence limit; LC90, 95% CI, lethal concentration causing 90% mortality and its 95% confidence interval; ppm, parts per million; χ2, heterogeneity about the regression line; df, degrees of freedom; NS, not significant.

^a^ h, heterogeneity factor, h = χ2/df If h < 1, the model provided a good fit to the data.

^b^ the *P* values less than 0.05 represented correlations between PREO doses and mortalities.

The four major components identified in PREO showed the larvicidal activity against BFs of *An*. *stephensi*, as well. The statistical analysis of the larval mortality for each component of the PREO is shown individually in the corresponding columns in Table 3. There were no statistically significant differences in the larval mortality between the PREO components at the dose of 11.44 ppm [F(4,15) = 1.29, P = 0.31]. However, at the concentration of 42.42 ppm, there was a significant difference between the PREO components [F(4,15) = 842.8, P≤0.001]. The results of post hoc tests (Tukey’s test) revealed a significant difference between citronellol, geraniol, and the mixture of four components (P≤0.05) and between the first two mentioned components and L-menthone (P≤0.05) with linalool. There was also a significant difference between the studied components at a dose of 12.55 ppm [F(4,15) = 9.60, P = 0.005], as well. The larvicidal activity of L-menthone and geraniol (P≤0.05), the mixture of four compounds (P≤0.01), and citronellol (P≤0.001) were significantly more than linalool. Statistically significant differences were found in the larval mortality between the components at the dose of 47.69 ppm [F(4,15) = 2227, P≤0.001]. According to Tukey’s results, all components and their mixture showed extremely higher larvicidal activity than linalool (P≤0.001).

**Table 3:** Mortality of larvae of two biological forms of *Anopheles stephensi* exposed to individual/mixed components of *Pelargonium roseum* essential oil based on the LC_50_ and LC_90_ concentrations.

	Mortality (% ± SD)					ANOVA test	
Biotype	Citronellol	L-menthone	Linalool	Geraniol	Mixture of four components	F(4,15)	*P* value
***An*. *stephensi mysorens***							
11.44 ppm	16.25±2.39	14.28±1.26	10.48±2.00	15.00±2.87	15.62±0.39	1.29	0.31
42.42 ppm	100±0.00***^,^^**###**^	27.67±2.63*	20.20±1.88	100±0.00***^,^^**###**^	100±0.00***^,^^**###**^	842.8	<0.001
***An*. *stephensi intermediate***							
12.55 ppm	46.05±0.77***	40.51±1.18*	27.47±1.12	40.59±4.81*	42.1±1.57**	9.60	0.005
47.69 ppm	100±0.00***	94.92±0.169***	38.75±1.25	100±0.00***	100±0.00***	2227	<0.001

Result Multiple Comparison (Tukey):

*P≤0.05,

** P≤0.01, and

*** P≤0.001 vs. linalool.

#P≤0.05,

## P≤0.01, and

### P≤0.001 vs. L-menthone.

The highest and the lowest larvicidal activity in both forms of *An*. *stephensi* were related to the citronellol and linalool, respectively. Details on the larvicidal activity of PREO and its main constituents are offered in Tables 2 and 3.

### Larvicidal activity of PREO nanoemulsions and selection of the optimum formulations

**Fig 2:**
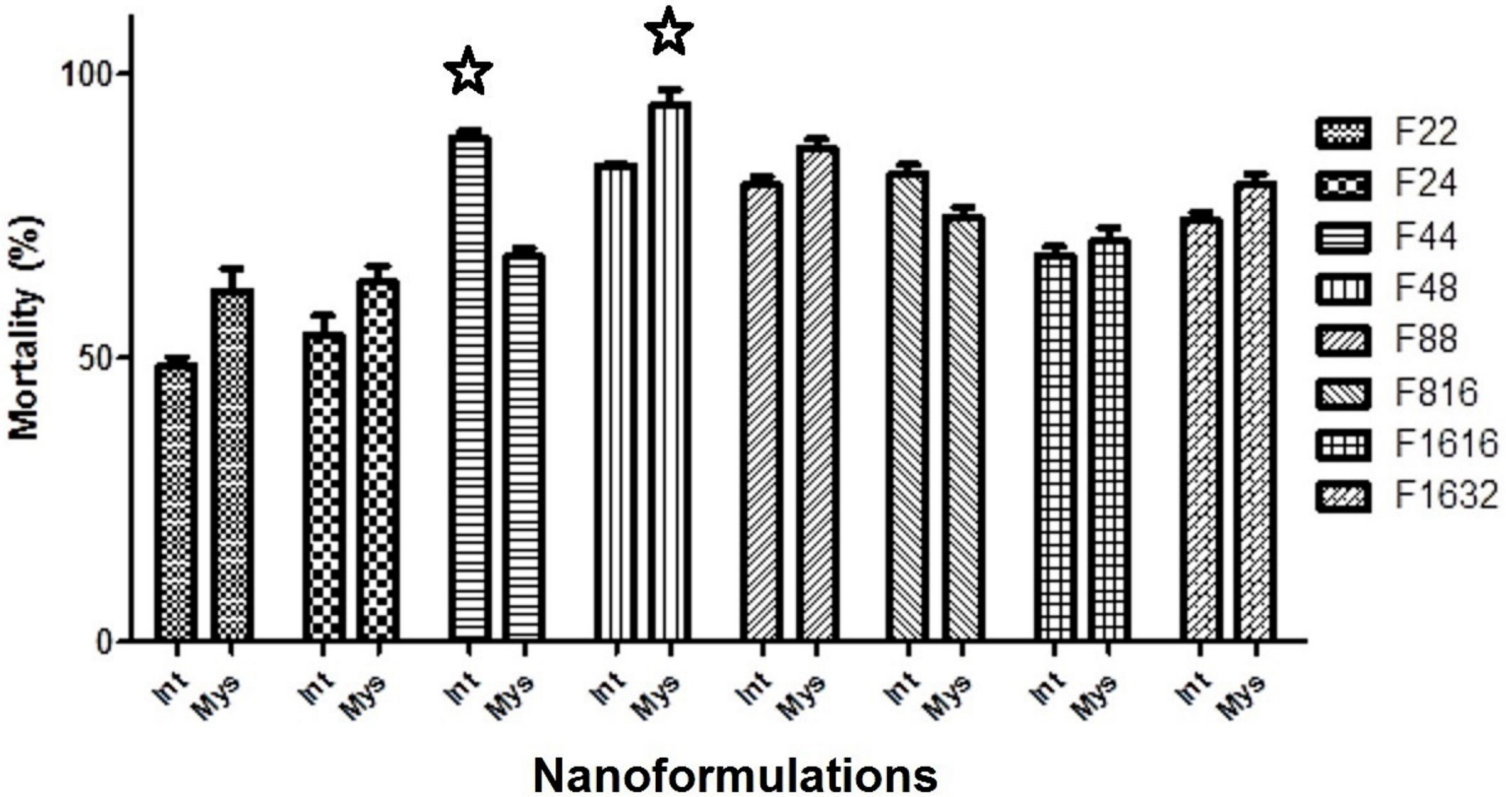
Larvicidal activities of nanoemulsions of *Pelargonium roseum* essential oil against mysorensis and intermediate forms of *Anopheles stephensi*. The most potent formulations are highlighted with stars.

Sixteen O/W formulations of PREO were freshly prepared, and their larvicidal activity was determined as explained before. Among the formulations, F48 and F44 showed the highest larvicidal effects against *An*. *stephensi mysorensis* and *An*. *stephensi intermediate*, respectively (Fig 2). The larvicidal activity of F48 was determined to be 94.44±2.680% in which 400 μL of TW, 800 μL of ETH, and 11.5 μL of PREO were applied. All nanoemulsions used against *An*. *stephensi mysorensis* generated mortality above 60% (values above calculated LC_50_). After F48, the second rank of the larvicidal activity was related to F88 with 86.93±1.533% lethality. In case of the *An*. *stephensi intermediate*, the average larvicidal activity of F44 was determined as 88.54±1.303%, followed by F48 with 83.75±0.417% lethality. With the exception of F22 and F24, other formulations accounted for more than 60% mortality of the larvae of *An*. *stephensi intermediate* form.

### Comparison of larvicidal activity of optimized nanoformulations with PREO

The larvicidal activity of the optimal nanoformulations affecting both BFs of *An*. *stephensi* was found to be twice more than that of PREO LC_50_ equivalent (P≤0.01; Table 4).

**Table 4:** Comparison of larvicidal activity of optimized nanoformulations with corresponding *Pelargonium roseum* essential oil LC_50_.

	*An*. *stephensi mysorensis*		*An*. *stephensi intermediate*	
Formulation	PREO (11.5 ppm)	F48	PREO (12.5 ppm)	F44
Mortality (%)	41.25±8.004**	94.44±2.268	39.59±6.091^##^	88.53±1.302

Results according to student’s t-test.

** P≤0.01 vs. F48 and

^##^ P≤0.01 vs. F44.

### Characterization of PREO nanoemulsions

**Fig 3:**
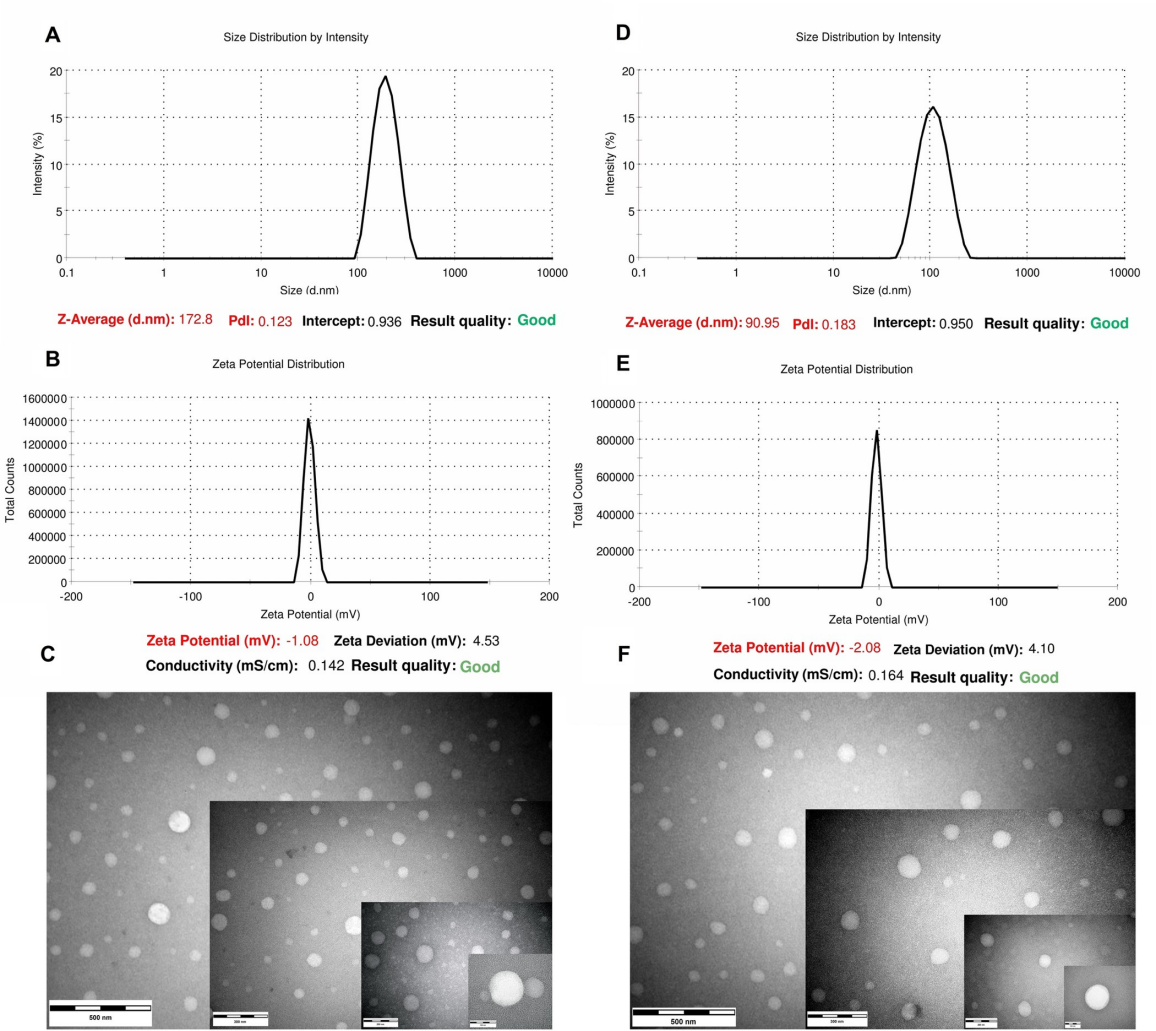
Physical properties and TEM micrographs of the optimized nanoemulsions; A, B, and C; for F48, D, E, and F; for F44.

**Fig 4:**
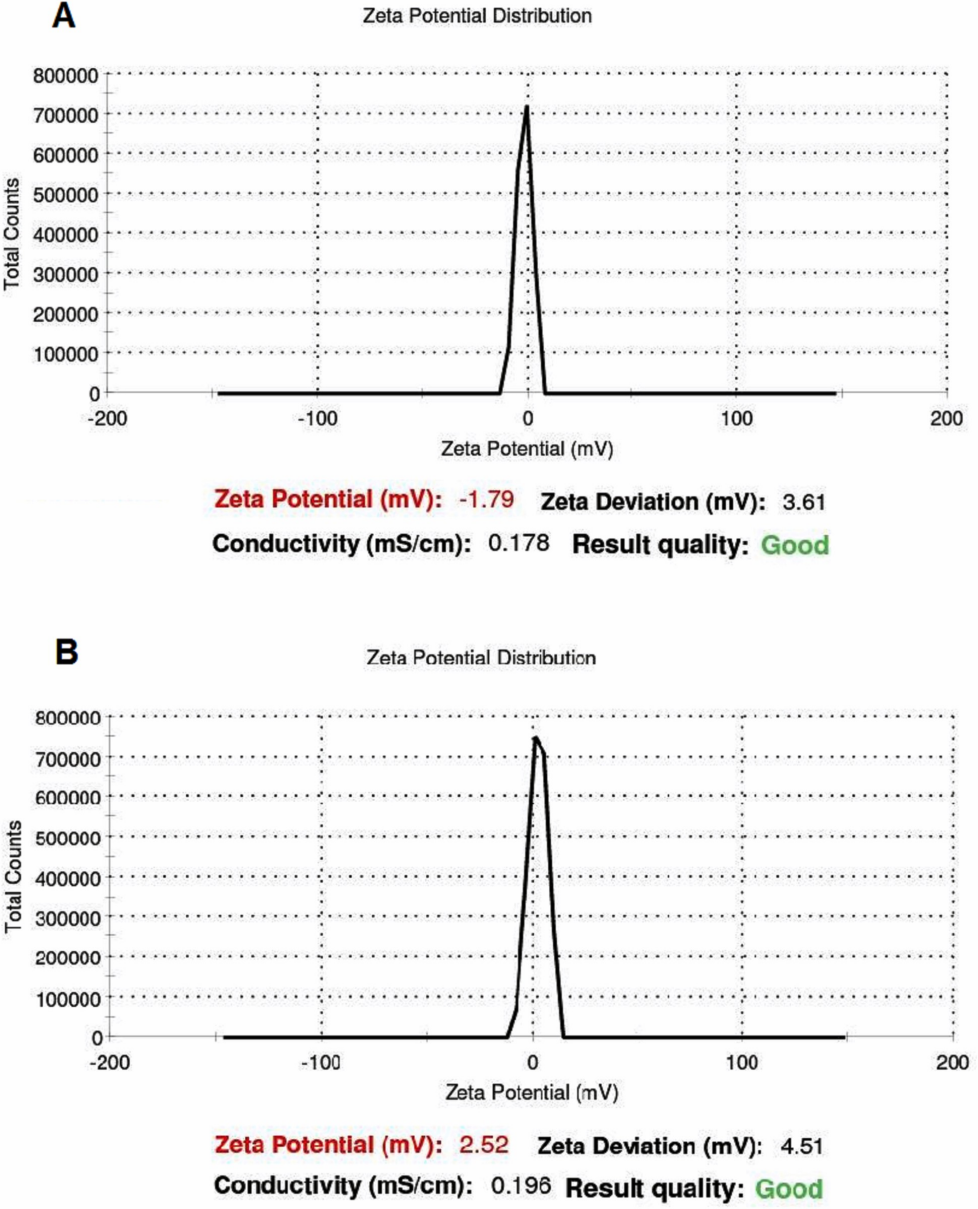
Zeta potential distribution of the optimized nanoemulsions after 40 days; (A) for F48, and (B) for F44.

The average PS of F48 and F44 nanoformulations was determined as 172.8 nm and 90.95 nm, and their PDI values were also found to be 0.123 and 0.183, respectively. The ZPs of the two particles were -1.08 mV and -2.08 mV, respectively (Fig 3A, 3B, 3D and 3E). TEM results revealed that the size of the sphere-shaped droplets was about 100 nm (Fig 3C and 3F), accordant with the DLS measures. After 40 days, the opacity of the F48 and F44 nanoemulsions did not change in the storage conditions; however, the ZPs were correspondingly raised to -1.79 and 2.52 (Fig 4). These data indicate that the stability of the prepared nanoemulsions have been maintained during this time.

### Interaction of larval aerobic gut bacteria with PREO and optimized nanoemulsions

*Serratia oryzae* and *Exiguobacterium profundum* were identified as the most abundant bacteria in the *An*. *stephensi mysorensis*. The predominant gut bacteria in the *An*. *stephensi intermediate* were *Acinetobacter junii* and *Bacillus pumilus*. Over 1400 bp of the *16S rDNA* sequences of the identified bacteria were successfully sequenced and deposited in the GenBank with the accession numbers MN197761-64. These representative bacteria were used individually or in combination to investigate the inhibitory effects of PREO formulations.

**Fig 5:**
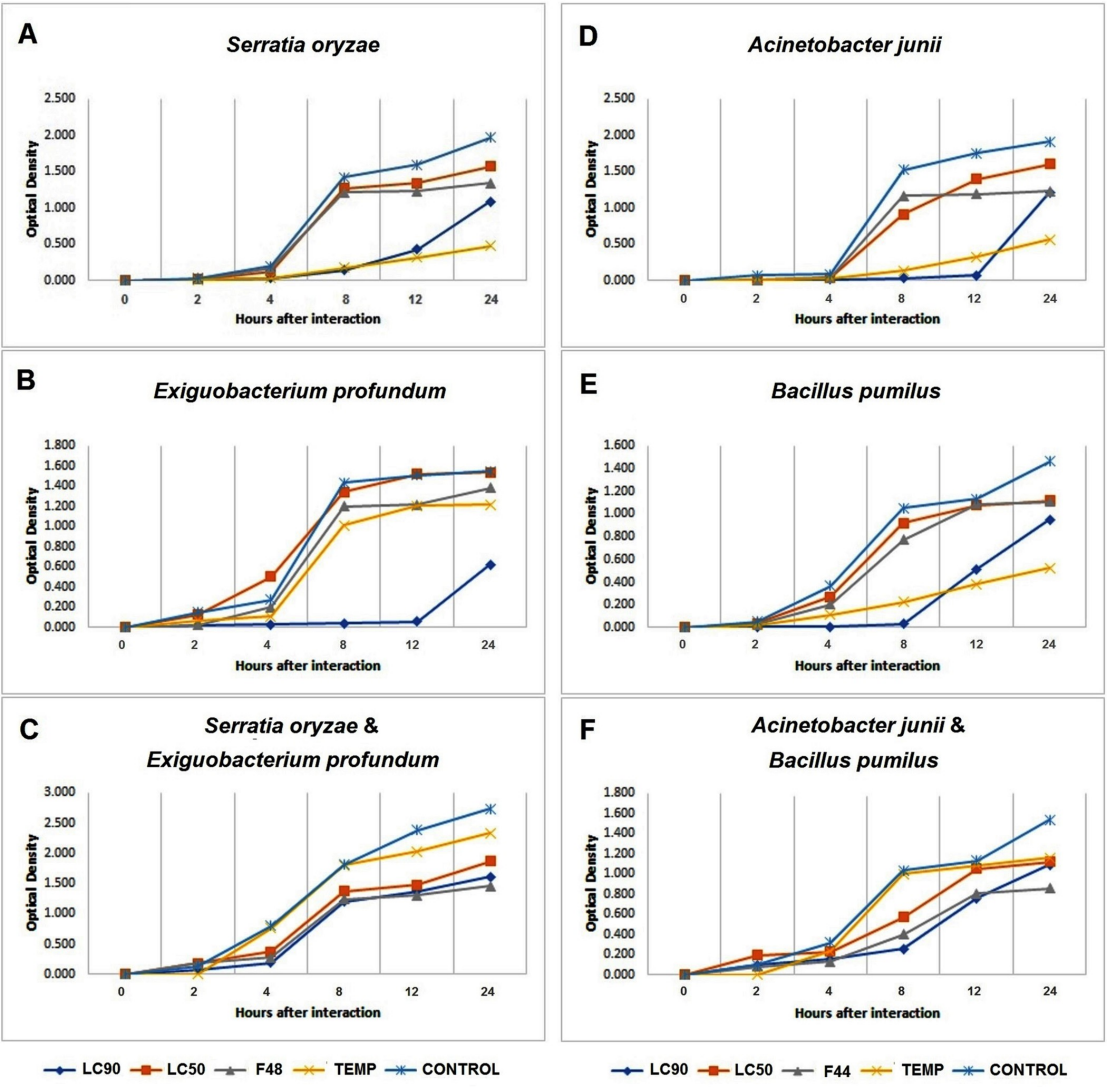
Growth of larval gut bacteria of *An*. *stephensi mysorensis* (left) and *An*. *stephensi intermediate* (right) in the presence of different concentrations of PREO (LC_50/90_) and optimized nanoformulations (F48/F44), as well as temephos. The untreated bacteria species were set as the control group. The absorbance measurement was taken at 600 nm at 2^th^, 4^th^, 8^th^, 12^th^, and 24^th^ time points during incubation at 37°C.

In general, the PREO and optimized nanoemulsions displayed potent antibacterial properties against the gut bacteria of the larvae of *An*. *stephensi* (Fig 5). In treatments with only one bacterium, the growth of bacteria was well suppressed for up to 4 hpi, then increased exponentially up to 8 hpi, and eventually became constant to some extents by the end of the interaction. However, in the presence of both Gram-positive and Gram-negative bacteria, the growth pattern occurred relatively in a linear manner (Fig 5).

PREO-based formulations indicated antibacterial activity against four bacteria species in the following order of decreasing inhibitory: LC_90_, optimized nanoemulsions, and LC_50_ (Table 5). Overall, the optimized formulations showed better antibacterial activity in the individual interaction mode compared to the LC_50_ and in the combined mode compared to the LC_50_ and temephos (P≤0.01). Also, in the combined interactions, the performance of the optimized formulations was similar to that of LC_90_. Details on the growth inhibition of bacteria exposed to different concentrations of PREO formulations are presented in the Table 5.

**Table 5:** Growth inhibition of bacteria exposed to different concentrations of *Pelargonium roseum* essential oil (LC_50/90_) and optimized nanoformulations (F48/F44), as well as temephos during 24 h.

Bacteria	Inhibitions of bacterial growth by formulations (% ± SD)				ANOVA test	
	LC_90_	LC_50_	F48/F44	Temephos	F(3,12)	*P* value
SO	67.20 ± 3.01	17.14 ± 1.17	24.86 ± 2.21	80.48 ± 27.95	551.18	<0.001
EP	84.19 ± 1.92	2.27 ± 0.63	18.05 ± 0.63	26.47 ± 1.16	34879.9	<0.001
SO & EP	43.43 ± 3.01	33.01 ± 2.11	43.16 ± 4.29	11.95 ± 0.92	106	<0.001
AJ	74.89 ± 2.97	26.10 ± 2.99	32.10 ± 3.54	79.87 ± 3.36	302.19	<0.001
BP	62.90 ± 4.33	15.99 ± 0.71	21.51 ± 0.76	68.87 ± 4.54	290.58	<0.001
AJ & BP	44.68 ± 3.73	23.31 ± 2.44	44.76 ± 1.12	15.60 ± 1.52	153.80	<0.001

SO: *Serratia oryzae*, EP: *Exiguobacterium profundum*, AJ: *Acinetobacter junii*, and BP: *Bacillus pumilus*.

Results from multiple comparisons (Tukey): *P≤0.05, ** P≤0.01, and *** P≤0.001:

**SO:** LC_90_ vs. LC_50_, F48/F44, and temephos***; LC_50_ vs. F48/F44**; LC_50_ vs. temephos***; F48/F44 vs. temephos***.

**EP:** LC_90_ vs. LC_50_, F48/F44, and temephos ***; LC_50_ vs. F48/F44 and temephos ***; F48/F44 vs. temephos***.

**SO & EP:** LC_90_ vs. LC_50_, and temephos***; LC_50_ vs. F48/F44**; LC_50_ vs. temephos***; F48/F44 vs. temephos***.

**AJ:** LC_90_ vs. LC_50_, and F48/F44 ***, LC_50_ vs. temephos***; F48/F44 vs. temephos***.

**BP:** LC_90_ vs. LC_50_ and F48/F44***; LC_50_ vs. temephos***; F48/F44 vs. temephos***.

**AJ & BP:** LC_90_ vs. LC_50_ and temephos ***; LC_50_ vs. F48/F44 and temephos **; F48/F44 vs. temephos***.

## Discussion

Herbal EOs, as green pesticides, have practically displayed insecticidal, fumigant, antifeedant, attractive, repellent, and growth-reducing effects on various arthropod pests, including insects of medical and veterinary significance [34, 74, 75]. Botanical pesticides made from EOs exploit the toxicity of aromatic hydrocarbons included in the oils [76]. This is an important advantage because these bioinsecticides affect only the target insects, do not damage beneficial natural enemies and provide residue-free food and safe environment [77]. As concluded in the literature, most of the EOs and their active substances are nontoxic to mammals. In general, their LD_50_ for rats have been reported to be 800–3,000 mg kg^-1^ for raw compounds and ≥5,000 mg kg^-1^ for formulated insecticides [78]. Thence most EOs and their active constituents can be used without toxicological and ecotoxicological studies [78]. Pelargoniums, with a vast variety of growth habits and habitats, are cultivated both for their beauty as ornamental plants and for their scent, as an important factor, in perfume, food, and beverages industries [79, 80].

Previous studies have shown the excito-repellency effects of PREO on adults *An*. *stephensi* [81] and on larvicidal activities against *Aedes aegypti* [82], *Culex pipiens* [83], and *Cx*. *quinquefasciatus* [84]. Nevertheless, its larvicidal impact on *An*. *stephensi*, which breeds in varied habitats, has not yet been investigated. The results of the current study disclosed the larvicidal effect of PREO and its major components on two BFs of *An*. *stephensi*. These findings are significant from two perspectives; first, the larvicidal activity of pure PREO is acceptable in comparison with the temephos, and second, the susceptibility of the two forms of *An*. *stephensi*, mysorensis and intermediate, was found to differ in terms of exposure to the oil. In this study, the PREO, even its major components, was more effective against *An*. *stephensi mysorensis* than *An*. *stephensi intermediate* (Fig 1 and Table 2). Moreover, the LC_50_ achieved an average of 12 ppm for both BFs studied. According to the categories suggested for the larvicidal activity of plant EOs [71], PREO can be categorized into the third class of active compounds, demanding for further consideration and investigation.

In agreement with other studies [83, 85–87], the major profiles of PREO were determined as citronellol, geraniol, linalool, and L-menthone by GC–MS analysis (Table 1). Citronellol, well-known as dihydro-geraniol, is the most abundant component of the oil. Geraniol, an acyclic monoterpene alcohol, is actually the main ingredient of citronellol synthesis in many plants. This compound is synthesized via ionization-dependent reaction or converted to citronellol by microbiological reduction [88]. Linalool is also a structural isomer of geraniol. L-menthone is a monoterpene with a minty flavor that occurs naturally in a number of EOs. With the available libraries, it was possible for us to accurately identify ~82% of the components in PREO, while the remaining 18%, which may contain the PREO metabolites, was not distinguished. Although the expected bioactivity was achieved in the identified components, inclusive libraries are required to determine unknown chemical compounds.

The bioactivities of the oxygenated monoterpenes, known in PREO, have been demonstrated in various surveys [88–92]. In our work, all the major components of PREO displayed the larvicidal activity; however, the highest larvicidal activity was related to citronellol and the least to linalool. In line with our results, former investigations have already been evidenced that both citronellol and geraniol are more toxic to *Cx*. *pipiens* and *Pediculus humanus capitis* than linalool [83, 85]. Lipophilic citronellol can disrupt the membrane integrity by inducing free radical generation [93]. Geraniol has also been considered as a penetration enhancer in transdermal drug delivery [94]. Likewise, geraniol can restore susceptibility to drugs in numerous Gram-negative bacteria by targeting efflux pumps [95].

Owing to their volatility, EOs have an easier degradation and cause less spread of environmental pollution. However, their solubility and stability problems need to be resolved in some ways, for example through the development of nanoformulations [96]. Accordingly, we developed 16 O/W nanoemulsions. Compared to the crude PREO, the overall larvicidal activity of all nanoformulations was boosted by 20% and the optimized formulations by 50% (Fig 2 and Table 4). F48 with ~94% and F44 with ~88% larval mortalities were nominated as the optimum formulations for *An*. *stephensi mysorensis* and *An*. *stephensi intermediate*, respectively (Fig 2). The physical properties of the optimized nanoformulations provided acceptable results under preparation conditions. Although there were no significant changes in the physical characteristics of the formulations kept for up to 40 days, future studies may examine their bioactivities, which were not the purpose of this study.

PREO is stable under normal temperature conditions, and the larvicidal assays of this study were performed under constant laboratory conditions; nonetheless, the effectiveness of EOs could be significantly influenced by post use temperature. This phenomenon has been noticed in the Pavela and Sedlak’s study [97] in which the effects of temperature on the insecticidal efficacy of an essential oil from *Thymus vulgaris*, in terms of acute toxicity against the larvae of *Spodoptera littoralis* and *Cx*. *quinquefasciatus*, have been explored. They reported that the lethal doses significantly decreased with rising/decreasing temperature against *S*. *littoralis*/*Cx*. *quinquefasciatus* larvae, respectively [97]. Therefore, the effects of ambient temperature on the larvicidal activity of PREO against *An*. *stephensi* forms, which are often found in sub-tropical regions, call for further investigation.

The results of this study showed the optimization of the PREO nanoformulations by applying different amounts of surfactant and co-surfactant. Dissimilarity between the optimal formulations affecting the two BFs may reflect the physiological and even genetic variations in the populations of the two studied BFs. *Anopheles stephensi* has not been regarded as a species complex in the literature; however, morphological differences in the number of egg ridges [69, 98], spiracular index [99], genetic variations in the intron I sequences of odorant-binding protein 1 [100], cytogenetic characteristics [101, 102], cuticular hydrocarbon profiles [103], and disparities in ecological, behavioral and mating characteristics [104], together with the findings of this study call into question this hypothesis.

The contributions of insect gut microbiota in the susceptibility and resistance to antimicrobials have recently been the focus of researchers [67, 105]. The function of gut bacteria against toxins can be protective or synergistic. Basically, insect gut bacteria are resistant to antimicrobials and to toxic ingredients of the food, or at least actively participate in detoxifying such compounds [106]. In this context, the antibacterial effects of PREO and its optimized nanoemulsions were investigated on representative gut bacteria of two BFs of *An*. *stephensi*. The whole PREO-based formulations displayed potent antibacterial activities against the symbiotic bacteria of the mysorensis and intermediate forms. However, the bacterial growth inhibition results varied depending on the species and Gram staining (Fig 5). Surprisingly, in the treatments with both Gram-positive and Gram-negative bacteria, all the PREO-based formulations uncovered a better inhibitory effect than temephos. This behavior may reflect the interaction of the bacteria under study, which encourages future studies to consider the sensitivity or resistance of the whole microbial community in the evaluation of antimicrobials. It should also be clarified in future investigations whether the antibacterial consequence of PREO inhibits the bacterial growth (bacteriostatic) or destroys bacterial cells (bactericidal), as well as determines the mechanisms underlying such actions.

EOs are active against a broad variety of organisms with multiple mechanisms, though their anti-insect mode of action can be categorized as behavioral and physiological. Behavioral mode of action will be true in adult insects in which volatile components of the oil can disrupt the communication behavior of insects by blocking the function of antennal sensilla [107]. The activities of some EOs appear to be the result of effects on the insect nervous system, either by the inhibition of acetylcholinesterase [108] or by the antagonism of the octopamine receptors [109]. The rapid action against some pests is indicative of a neurotoxic mode of action. The lack of octopamine receptors in vertebrates provides the mammalian selectivity of EOs as insecticides [110, 111]. Therefore, plant EOs and their components can influence the physiological functions of different insect species and modulate their gut microbiota, as the results of the present study indicated.

In the present research, the antibacterial performance of PREO nanoemulsions was comparable with temephos, and this type of performance may be attributable to their antibacterial activity; however, further (proteomic and enzymatic) studies are needed to identify the inhibitors of enzymes involved in susceptibility/resistance to toxic compounds.

As a non-systemic organophosphorus insecticide, temephos has extensively been used to treat water infested with disease-carrying insects, including mosquitoes, midges, and black fly larvae. It has also been recommended for the treatment of drinking-water sources and containers [112, 113]. Regrettably, resistance (and tolerance in this study) to temephos has been detected in the *Culex* spp. [114, 115], *Aedes* spp. [116–118], and *An*. *stephensi* [119, 120]. This issue sounds the alarm for using temephos in Iran and around the world.

## Conclusion

The results of this study approved the larvicidal and antibacterial effects of pure PREO and its nanoformulations on two BFs of *An*. *stephensi* and their intestinal bacteria. Difference between the PREO-based formulations affecting the two BFs may reflect the physiological and even genetic variations in the populations of *An*. *stephensi* studied. The larvicidal activity of PREO-based formulations can ascribed to their antibacterial activity, which calls for further investigation. The susceptibility or resistance of the intestinal bacterial flora may determine the outcomes of the pesticides effects on the target insects. This situation affirms the potential of the bacteria as a major contributor to the survival or inexistence of insects. The formulations developed herein are cost-effective and possibly will have the least damage to humans, environment, and non-target organisms and would be comparable to industrial larvicides, e.g. temephos, if they pass semi-field and field trials successfully.

## References

[1] Lee H, Halverson S, Ezinwa N. Mosquito‐borne diseases. Prim Care Clin off Pract. 2018; 45:393–407. 10.1016/j.pop.2018.05.001 30115330

[2] WHO. World malaria report 2019. World Health Organization; 2019. https://www.who.int/malaria/publications/world-malaria-report-2019/en/

[3] Hay SI, Sinka ME, Okara RM, Kabaria CW, Mbithi PM, Tago CC, et al Developing global maps of the dominant Anopheles vectors of human malaria. PLoS med. 2010; 7: e1000048 10.1371/journal.pmed.1000209 20161718 PMC2817710

[4] Manouchehri A, Javadian E, Eshighy N, Motabar M. Ecology of *Anopheles stephensi* Liston in southern Iran. Trop Geogr Med. 1976; 28:228–232. 1006792

[5] Vatandoost H, Oshaghi M, Abaie M, Shahi M, Yaaghoobi F, Baghaii M, et al Bionomics of *Anopheles stephensi* Liston in the malarious area of Hormozgan province, southern Iran, 2002. Acta Trop. 2006; 97:196–203. 10.1016/j.actatropica.2005.11.002 16329986

[6] Karimian F, Oshaghi MA, Sedaghat MM, Waterhouse RM, Vatandoost H, Hanafi-Bojd AA, et al Phylogenetic analysis of the oriental-Palearctic-Afrotropical members of Anopheles (Culicidae: Diptera) based on nuclear rDNA and mitochondrial DNA characteristics. Jap J Infec Dis. 2014; 67:361–367. 10.7883/yoken.67.361 25241686

[7] Hoosh-Deghati H, Dinparast-Djadid N, Moin-Vaziri V, Atta H, Raz AA, Seyyed-Tabaei SJ, et al Composition of Anopheles species collected from selected Malarious areas of Afghanistan and Iran. J Arthropod Borne Dis. 2017; 11: 354–362. 29322052 PMC5758631

[8] Krishnan KS. Anophele stephensi Liston 1901. Vectors of Malaria in India 2nd ed Delhi: National Society of India for Malaria and other Mosquito-borne Disease. 1961.

[9] Sinka ME, Bangs MJ, Manguin S, Chareonviriyaphap T, Patil AP, Temperley WH, et al The dominant Anopheles vectors of human malaria in the Asia-Pacific region: occurrence data, distribution maps and bionomic précis. Parasit Vectors. 2011; 4:89 10.1186/1756-3305-4-89 21612587 PMC3127851

[10] Oshaghi M, Yaghoobi F, Vatandoost H, Abai M, Akbarzadeh K. *Anopheles stephensi* biological forms, geographical distribution, and malaria transmission in malarious regions in Iran. Pak J Biol Sci. 2006; 9: 294–298.

[11] Chavshin AR, Oshaghi MA, Vatandoost H, Hanafi-Bojd AA, Raeisi A, Nikpoor F. Molecular characterization, biological forms and sporozoite rate of Anopheles stephensi in southern Iran. Asian Asian Pac J Trop Biomed. 2014; 4:47–51. 10.1016/S2221-1691(14)60207-0 24144130 PMC3819495

[12] Matsuoka H, Yoshida S, Hirai M, Ishii A. A rodent malaria, *Plasmodium berghei*, is experimentally transmitted to mice by merely probing of infective mosquito, *Anopheles stephensi*. Parasitol Int. 2002; 51:17–23. 10.1016/s1383-5769(01)00095-2 11880224

[13] Zaini A, Djanbakhsh B, Manuchehri A. Characteristics of breeding places of Anopheles stephensi in a city on persian gulf. Iran J Public Health. 1975;114–118.

[14] Hanafi-Bojd A, Vatandoost H, Oshaghi M, Charrahy Z, Haghdoost A, Sedaghat M, et al Larval habitats and biodiversity of Anopheline mosquitoes (Diptera: Culicidae) in a malarious area of southern Iran. J Vector Borne Dis. 2012; 49:91 22898481

[15] Niang EHA, Bassene H, Fenollar F, Mediannikov O. Biological Control of Mosquito-Borne Diseases: The Potential of Wolbachia-Based Interventions in an IVM Framework. J Trop Med. 2018; 1470459 10.1155/2018/1470459 30581476 PMC6276417

[16] Mulla M. Mosquito control then, now, and in the future. J Am Mosq Control Assoc. 1994; 10:574–84. 7707066

[17] Tusting LS, Thwing J, Sinclair D, Fillinger U, Gimnig J, Bonner KE, et al Mosquito larval source management for controlling malaria. Cochrane Database Syst Rev. 2013; 8: CD008923 10.1002/14651858.CD008923.pub2 23986463 PMC4669681

[18] WHO. Handbook for Integrated Vector Management. WHO Press, Geneva, Switzerland 2013.

[19] Bird S, Traub S, Grayzel J. Organophosphate and carbamate poisoning. UpToDate. 2014; 14:339.

[20] Srivastava AK, Kesavachandran C. Health Effects of Pesticides. New Delhi: The Energy and Resources Institute; 2016.

[21] Sougoufara S, Doucouré S, Sembéne PMB, Harry M, Sokhna C. Challenges for malaria vector control in sub-Saharan Africa: resistance and behavioral adaptations in Anopheles populations. J Vector Borne Dis. 2017; 54:4 28352041

[22] Zaim M, Aitio A, Nakashima N. Safety of pyrethroid‐treated mosquito nets. Med Vet Entomol. 2000; 14:1–5. 10.1046/j.1365-2915.2000.00211.x 10759305

[23] Chrustek A, Hołyńska-Iwan I, Dziembowska I, Bogusiewicz J, Wróblewski M, Cwynar A, et al Current research on the safety of pyrethroids used as insecticides. Medicina. 2018; 54:61 10.3390/medicina54040061 30344292 PMC6174339

[24] Collotta M, Bertazzi P, Bollati V. Epigenetics and pesticides. Toxicology. 2013; 307:35–41. 10.1016/j.tox.2013.01.017 23380243

[25] Aktar W, Sengupta D, Chowdhury A. Impact of pesticides use in agriculture: their benefits and hazards. Interdiscip Toxicol. 2009; 2:1–12. 10.2478/v10102-009-0001-7 21217838 PMC2984095

[26] Roubos CR, Rodriguez-Saona C, Isaacs R. Mitigating the effects of insecticides on arthropod biological control at field and landscape scales. Biol Control. 2014; 75:28–38.

[27] Özkara A, Akyıl, D., & Konuk, M Pesticides, environmental pollution, and health. Environmental health risk hazardous factors to living species Rijeka: Publisher: InTech. 2016.

[28] Karunamoorthi K, Sabesan S. Insecticide resistance in insect vectors of disease with special reference to mosquitoes: a potential threat to global public health. 2013.

[29] Benelli G, Jeffries CL, Walker T. Biological control of mosquito vectors: past, present, and future. Insects. 2016; 7:52. 10.3390/insects7040052 PMC5198200 27706105

[30] Coutinho-Abreu IV, Zhu KY, Ramalho-Ortigao M. Transgenesis and paratransgenesis to control insect-borne diseases: current status and future challenges. Parasitol Int. 2010; 59:1–8. 10.1016/j.parint.2009.10.002 19819346 PMC2824031

[31] Maleki-Ravasan N, Oshaghi MA, Afshar D, Arandian MH, Hajikhani S, Akhavan AA, et al Aerobic bacterial flora of biotic and abiotic compartments of a hyperendemic Zoonotic Cutaneous Leishmaniasis (ZCL) focus. Parasit Vectors. 2015; 8:63 10.1186/s13071-014-0517-3 25630498 PMC4329651

[32] Dehghan H, Oshaghi MA, Moosa-Kazemi SH, Yakhchali B, Vatandoost H, Maleki-Ravasan N, et al Dynamics of transgenic Enterobacter cloacae expressing green fluorescent protein defensin (GFP-D) in Anopheles stephensi under laboratory condition. J Arthropod Borne Dis. 2017; 11:515 29367928 PMC5775158

[33] Karimian F, Vatandoost H, Rassi Y, Maleki-Ravasan N, Mohebali M, Shirazi MH, et al Aerobic midgut microbiota of sand fly vectors of zoonotic visceral leishmaniasis from northern Iran, a step toward finding potential paratransgenic candidates. Parasit Vectors. 2019; 12:1–12. 10.1186/s13071-018-3256-z 30616668 PMC6322272

[34] Mossa A-TH. Green pesticides: Essential oils as biopesticides in insect-pest management. J Environ Sci Technol. 2016; 9:354.

[35] Tahghighi A, Maleki-Ravasan N, Djadid ND, Alipour H, Ahmadvand R, Karimian F, et al GC–MS analysis and anti–mosquito activities of Juniperus virginiana essential oil against Anopheles stephensi (Diptera: Culicidae). Asian Pac J Trop Biomed. 2019; 9:168.

[36] Nollet LMR H.S.. Green Pesticides Handbook: Essential Oils for Pest Control. CRC Press: Boca Raton, FL, USA 2017.

[37] Swamy MK, Akhtar MS, Sinniah UR. Antimicrobial properties of plant essential oils against human pathogens and their mode of action: an updated review. Evid Based Complement Alternat Med. 2016; 2016. 10.1155/2016/3012462 PMC5206475 28090211

[38] Kumar Y, Prakash O, Tripathi H, Tandon S, Gupta MM, Rahman L-U, et al AromaDb: A database of medicinal and aromatic plant’s aroma molecules with phytochemistry and therapeutic potentials. Front Plant Sci. 2018; 9:1081 10.3389/fpls.2018.01081 30150996 PMC6099104

[39] Dhifi W, Bellili S, Jazi S, Bahloul N, Mnif W. Essential oils’ chemical characterization and investigation of some biological activities: a critical review. Medicines. 2016; 3:25 10.3390/medicines3040025 28930135 PMC5456241

[40] Bilia AR, Guccione C, Isacchi B, Righeschi C, Firenzuoli F, Bergonzi MC. Essential oils loaded in nanosystems: a developing strategy for a successful therapeutic approach. Evid Based Complement Altern Med. 2014; 2014:651593 10.1155/2014/651593 24971152 PMC4058161

[41] Odriozola-Serrano I, Oms-Oliu G, Martín-Belloso O. Nanoemulsion-based delivery systems to improve functionality of lipophilic components. Front Nutr. 2014; 1:24 10.3389/fnut.2014.00024 25988126 PMC4428376

[42] Campolo O, Giunti G, Russo A, Palmeri V, Zappalà L. Essential oils in stored product insect pest control. J Food Qual 2018:1–18.

[43] Giunti G, Palermo D, Laudani F, Algeri GM, Campolo O. and Palmeri V. Repellence and acute toxicity of a nano-emulsion of sweet orange essential oil toward two major stored grain insect pests. Ind Crop Prod. 2019; 142: 111869.

[44] Sugumar S, Clarke S, Nirmala M, Tyagi B, Mukherjee A, Chandrasekaran N. Nanoemulsion of eucalyptus oil and its larvicidal activity against *Culex quinquefasciatus*. Bull Entomol Res. 2014; 104:393–402. 10.1017/S0007485313000710 24401169

[45] Duarte JL, Amado JR, Oliveira AE, Cruz RA, Ferreira AM, Souto RN, et al Evaluation of larvicidal activity of a nanoemulsion of Rosmarinus officinalis essential oil. Rev Bras Farmacogn. 2015; 25:189–92.

[46] Oliveira AE, Duarte JL, Amado JR, Cruz RA, Rocha CF, Souto RN, et al Development of a larvicidal nanoemulsion with *Pterodon emarginatus* Vogel oil. PLoS One. 2016; 11: 0145835 10.1371/journal.pone.0145835 26742099 PMC4711774

[47] Balasubramani S, Rajendhiran T, Moola AK, Diana RKB. Development of nanoemulsion from Vitex negundo L. essential oil and their efficacy of antioxidant, antimicrobial and larvicidal activities (Aedes aegypti L.). Environ Sci Pollut Res Int. 2017; 24:15125–33. 10.1007/s11356-017-9118-y 28497330

[48] Botas GdS, Cruz RA, De Almeida FB, Duarte JL, Araújo RS, Souto RNP, et al Baccharis reticularia DC. and limonene nanoemulsions: promising larvicidal agents for Aedes aegypti (Diptera: Culicidae) control. Molecules. 2017; 22:1990 10.3390/molecules22111990 29149027 PMC6150371

[49] Jesus FL, de Almeida FB, Duarte JL, Oliveira AE, Cruz RA, Souto RN, et al Preparation of a nanoemulsion with Carapa guianensis aublet (Meliaceae) oil by a low-energy/solvent-free method and evaluation of its preliminary residual larvicidal activity. Evid Based Complement Alternat Med. 2017; 6756793. 10.1155/2017/6756793 28798803 PMC5535731

[50] Osanloo M, Amani A, Sereshti H, Abai MR, Esmaeili F, Sedaghat MM. Preparation and optimization nanoemulsion of Tarragon (Artemisia dracunculus) essential oil as effective herbal larvicide against Anopheles stephensi. Ind Crops Prod. 2017; 109:214–219.

[51] Osanloo M, Sereshti H, Sedaghat MM, Amani A. Nanoemulsion of Dill essential oil as a green and potent larvicide against Anopheles stephensi. Environ Sci Pollut. 2018; 25:6466–6473. 10.1007/s11356-017-0822-4 29250730

[52] Sogan N, Kapoor N, Kala S, Patanjali P, Nagpal B, Kumar V, et al Larvicidal activity of castor oil Nanoemulsion against malaria vector Anopheles culicifacies. Int J Mosq Res. 2018; 5:01–06.

[53] Sundararajan B, Moola AK, Vivek K, Kumari BR. Formulation of nanoemulsion from leaves essential oil of Ocimum basilicum L. and its antibacterial, antioxidant and larvicidal activities (Culex quinquefasciatus). Microb Pathog. 2018; 125:475–485. 10.1016/j.micpath.2018.10.017 30340015

[54] Douglas AE. Multiorganismal insects: diversity and function of resident microorganisms. nnu Rev Entomol. 2015; 60:17–34. 10.1146/annurev-ento-010814-020822 25341109 PMC4465791

[55] Douglas AE. The microbial dimension in insect nutritional ecology. Funct Ecol. 2009; 23:38–47.

[56] Otti O. Genitalia‐associated microbes in insects. Insect Sci. 2015; 22:325–339. 10.1111/1744-7917.12183 25388748

[57] Karami M, Moosa-Kazemi SH, Oshaghi MA, Vatandoost H, Sedaghat MM, Rajabnia R, et al Wolbachia endobacteria in natural populations of Culex pipiens of Iran and its phylogenetic congruence. J Arthropod Borne Dis. 2016; 10: 349–365. 27308293 PMC4906741

[58] Brucker RM, Bordenstein SR. Speciation by symbiosis. Trends Ecol Evol. 2012; 27:443–451. 10.1016/j.tree.2012.03.011 22541872

[59] Russell RJ, Scott C, Jackson CJ, Pandey R, Pandey G, Taylor MC, et al The evolution of new enzyme function: lessons from xenobiotic metabolizing bacteria versus insecticide‐resistant insects. Evol Appl. 2011; 4:225–248. 10.1111/j.1752-4571.2010.00175.x 25567970 PMC3352558

[60] Sant’Anna MR, Diaz-Albiter H, Aguiar-Martins K, Al Salem WS, Cavalcante RR, Dillon VM, et al Colonisation resistance in the sand fly gut: *Leishmania* protects *Lutzomyia longipalpis* from bacterial infection. Parasit Vectors. 2014; 7:329 10.1186/1756-3305-7-329 25051919 PMC4112039

[61] Rodgers FH, Gendrin M, Wyer CA, Christophides GK. Microbiota-induced peritrophic matrix regulates midgut homeostasis and prevents systemic infection of malaria vector mosquitoes. PLoS Pathog. 2017; 13:e1006391 10.1371/journal.ppat.1006391 28545061 PMC5448818

[62] Maleki‐Ravasan N, Akhavan N, Raz A, Jafari M, Zakeri S, Dinparast Djadid N. Co‐occurrence of pederin‐producing and Wolbachia endobacteria in Paederus fuscipes Curtis, 1840 (Coleoptera: Staphilinidae) and its evolutionary consequences. MicrobiologyOpen. 2019; 8:e00777. 10.1002/mbo3.777 PMC6612549 30560551

[63] Noman A, Aqeel M, Qasim M, Haider I, Lou Y. Plant-insect-microbe interaction: A love triangle between enemies in ecosystem. Sci Total Environ. 2020; 699:134181 10.1016/j.scitotenv.2019.134181 31520944

[64] Weiss B, Aksoy S. Microbiome influences on insect host vector competence. Trends Parasitol. 2011; 27:514–522. 10.1016/j.pt.2011.05.001 21697014 PMC3179784

[65] Pietri JE, Tiffany C, Liang D. Disruption of the microbiota affects physiological and evolutionary aspects of insecticide resistance in the German cockroach, an important urban pest. PloS one. 2018; 13: e0207985 10.1371/journal.pone.0207985 30540788 PMC6291076

[66] Xia X, Sun B, Gurr GM, Vasseur L, Xue M, You M. Gut microbiota mediate insecticide resistance in the diamondback moth, Plutella xylostella (L.). Front Microbiol. 2018; 9:25 10.3389/fmicb.2018.00025 29410659 PMC5787075

[67] Barnard K, Jeanrenaud AC, Brooke BD, Oliver SV. The contribution of gut bacteria to insecticide resistance and the life histories of the major malaria vector Anopheles arabiensis (Diptera: Culicidae). Sci Rep. 2019; 9:1–11. 10.1038/s41598-018-37186-2 31235803 PMC6591418

[68] MR4. Methods in Anopheles research manual. Malaria Research and Reference Reagent Resource Centre; 2014. Available at (https://www.beiresources.org/Portals/2/PDFS/2014MethodsinAnophelesResearch ManualFullVersionv2tso.pdf, accessed 12 September 2016).

[69] Subbarao SK, Vasantha K, Adak T, Sharma V, Curtis C. Egg‐float ridge number in *Anopheles stephensi*: ecological variation and genetic analysis. Med Vet Entomol. 1987; 1:265–271. 10.1111/j.1365-2915.1987.tb00353.x 2979540

[70] WHO. Guidelines for laboratory and field testing of mosquito larvicides. World Health Organization; 2005.

[71] Vatandoost H, Dehkordi AS, Sadeghi S, Davari B, Karimian F, Abai M, et al Identification of chemical constituents and larvicidal activity of Kelussia odoratissima Mozaffarian essential oil against two mosquito vectors Anopheles stephensi and Culex pipiens (Diptera: Culicidae). Exp Parasitol. 2012; 132:470–474. 10.1016/j.exppara.2012.09.010 23022522

[72] Abbott WS. A method of computing the effectiveness of an insecticide. J Am Mosq Control Assoc.1925; 2, 302–303. 3333059

[73] Lei C, Sun X. Comparing lethal dose ratios using probit regression with arbitrary slopes. BMC Pharmacol Toxicol. 2018; 19, 1–10. 10.1186/s40360-017-0192-z 30290834 PMC6173863

[74] Koul O, Walia S, Dhaliwal G. Essential oils as green pesticides: potential and constraints. Biopestic Int. 2008; 4:63–84.

[75] Regnault-Roger C, Vincent C, Arnasson JT. Essential oils in insect control: low-risk products in a high-stakes world. Annu Rev Entomol. 2012; 57:405–425. 10.1146/annurev-ento-120710-100554 21942843

[76] Pavela R, Benelli G. Essential oils as eco-friendly biopesticides? Challenges and constraints. Trends Plant Sci. 2016; 21:1000–1007. 10.1016/j.tplants.2016.10.005 27789158

[77] Hikal WM, Baeshen RS, and Said-Al Ahl HAH. Botanical insecticide as simple extractives for pest control. Cogent Biology 2017; 3:1404274.

[78] Isman MB, Miresmailli S, Machial C. Commercial opportunities for pesticides based on plant essential oils in agriculture, industry and consumer products. Phytochem Rev. 2011;10: 197–204.

[79] Charlwood B, Charlwood K. Pelargonium spp.(Geranium): in vitro culture and the production of aromatic compounds. Medicinal and Aromatic Plants III: Springer; 1991 p. 339–352.

[80] Adams J AC, Barclay G, Barrow JH, Belsinger S, Brobst J, Cole J, et al. A Pelargoniums, An Herb Society of America Guide. The Herb Society of America 9019 Kirtland Chardon Rd Kirtland, Ohio. 2006.

[81] Alipour H, Mahdian SMA, Rami A, Abad MOK, Amin M, Dinparast N. Excito-repellency effects of Pelargonium roseum wild (Geraniaceae) essential oil-treated bed nets on the malaria mosquito, Anopheles stephensi Liston, 1901 (Diptera: Culicidae). J Entomol Zool Stud. 2015; 3:87–91.

[82] Amer A, Mehlhorn H. Larvicidal effects of various essential oils against Aedes, Anopheles, and Culex larvae (Diptera, Culicidae). Parasitol Res. 2006; 99:466–472. 10.1007/s00436-006-0182-3 16642386

[83] Tabari MA, Youssefi MR, Esfandiari A, Benelli G. Toxicity of β-citronellol, geraniol and linalool from Pelargonium roseum essential oil against the West Nile and filariasis vector Culex pipiens (Diptera: Culicidae). Res Vet Sci. 2017; 114:36–40. 10.1016/j.rvsc.2017.03.001 28297637

[84] Benelli G, Pavela R, Canale A, Cianfaglione K, Ciaschetti G, Conti F, et al Acute larvicidal toxicity of five essential oils (Pinus nigra, Hyssopus officinalis, Satureja montana, Aloysia citrodora and Pelargonium graveolens) against the filariasis vector Culex quinquefasciatus: Synergistic and antagonistic effects. Parasitol Int. 2017; 66:166–171. 10.1016/j.parint.2017.01.012 28110082

[85] Gallardo A, Picollo MI, González-Audino P, Mougabure-Cueto G. Insecticidal activity of individual and mixed monoterpenoids of geranium essential oil against Pediculus humanus capitis (Phthiraptera: Pediculidae). J Med Entomol. 2012; 49:332–325. 10.1603/me11142 22493851

[86] Essid R, Hammami M, Gharbi D, Karkouch I, Hamouda TB, Elkahoui S, et al Antifungal mechanism of the combination of *Cinnamomum verum* and *Pelargonium graveolens* essential oils with fluconazole against pathogenic Candida strains. Appl Microbiol Biotechnol. 2017; 101:6993–7006. 10.1007/s00253-017-8442-y 28766033

[87] El-Kareem M, A Rabbih M, Elansary HO, A Al-Mana F. Mass Spectral Fragmentation of Pelargonium graveolens Essential Oil Using GC–MS Semi-Empirical Calculations and Biological Potential. Processes. 2020; 8:128.

[88] Chen W, Viljoen A. Geraniol-a review of a commercially important fragrance material. S Afr J Bot. 2010; 76:643–651.

[89] Müller GC, Junnila A, Kravchenko VD, Revay EE, Butler J, Schlein Y. Indoor protection against mosquito and sand fly bites: a comparison between citronella, linalool, and geraniol candles. J Am Mosq Control Assoc. 2008; 24:150–153. 10.2987/8756-971X(2008)24[150:IPAMAS]2.0.CO;2 18437831

[90] Pereira FdO, Mendes JM, Lima IO, Mota KSdL, Oliveira WAd, Lima EdO. Antifungal activity of geraniol and citronellol, two monoterpenes alcohols, against Trichophyton rubrum involves inhibition of ergosterol biosynthesis. Pharm Biol. 2015; 53:228–234. 10.3109/13880209.2014.913299 25414073

[91] Guimarães AC, Meireles LM, Lemos MF, Guimarães MCC, Endringer DC, Fronza M, et al Antibacterial activity of terpenes and terpenoids present in essential oils. Molecules. 2019; 24:2471 10.3390/molecules24132471 31284397 PMC6651100

[92] Luna EC, Luna IS, Scotti L, Monteiro AFM, Scotti MT, de Moura RO, et al Active essential oils and their components in use against neglected diseases and arboviruses. Oxid Med Cell Longev. 2019; 2019:6587150 10.1155/2019/6587150 30881596 PMC6387720

[93] Kaur S, Rana S, Singh HP, Batish DR, Kohli RK. Citronellol disrupts membrane integrity by inducing free radical generation. Z Naturforsch C J Biosci. 2011; 66:260–266. 10.1515/znc-2011-5-609 21812343

[94] Aqil M, Ahad A, Sultana Y, Ali A. Status of terpenes as skin penetration enhancers. Drug Discov Today. 2007; 12:1061–1067. 10.1016/j.drudis.2007.09.001 18061886

[95] Lorenzi V, Muselli A, Bernardini AF, Berti L, Pagès J-M, Amaral L, et al Geraniol restores antibiotic activities against multidrug-resistant isolates from gram-negative species. Antimicrob Agents Chemother. 2009; 53:2209–2211. 10.1128/AAC.00919-08 19258278 PMC2681508

[96] Echeverría J, Albuquerque RDDGd. Nanoemulsions of essential oils: New tool for control of vector-Borne diseases and in vitro effects on some parasitic agents. Medicines. 2019; 6:42 10.3390/medicines6020042 30934720 PMC6630918

[97] Pavela R, Sedlak P. Post-application temperature as a factor influencing the insecticidal activity of essential oil from *Thymus vulgaris*. Ind Crop Prod. 2018; 113: 46–49.

[98] Sweet W, Rao B. Races of A. stephensi Liston, 1901. Ind Med Gaz. 1937; 72:665 29013249 PMC5173639

[99] Nagpal B, Srivastava A, Kalra N, Subbarao S. Spiracular indices in *Anopheles stephensi*: a taxonomic tool to identify ecological variants. J Med Entomol. 2003; 40:747–749. 10.1603/0022-2585-40.6.747 14765648

[100] Firooziyan S, Djadid ND, Gholizadeh S. Speculation on the possibility for introducing *Anopheles stephensi* as a species complex: preliminary evidence based on odorant binding protein 1 intron I sequence. Malaria journal. 2018; 17:1–7. 10.1186/s12936-017-2149-5 30326917 PMC6191895

[101] Coluzzi M, Di Deco M, Cancrini G. Chromosomal inversions in *Anopheles stephensi*. Parassitologia. 1973; 15:129–136. 4788354

[102] Suguna S. Y‐chromosome dimorphism in the malaria vector *Anopheles stephensi* from south India. Med Vet Entomol. 1992; 6:84–86. 10.1111/j.1365-2915.1992.tb00040.x 1600233

[103] Anyanwu G, Davies D, Molyneux D, Phillips A, Milligan P. Cuticular hydrocarbon discrimination/variation among strains of the mosquito, Anopheles (Cellia) stephensi Liston. Ann Trop Med Parasitol. 1993; 87:269–275. 10.1080/00034983.1993.11812766 8257238

[104] WHO. Anopheline species complexes in south and South-East Asia. SEARO technical publication 2007; 57:79–83.

[105] Dada N, Sheth M, Liebman K, Pinto J, Lenhart A. Whole metagenome sequencing reveals links between mosquito microbiota and insecticide resistance in malaria vectors. Sci Rep. 2018; 8:2084 10.1038/s41598-018-20367-4 29391526 PMC5794770

[106] Ignasiak K, Maxwell A. Antibiotic-resistant bacteria in the guts of insects feeding on plants: prospects for discovering plant-derived antibiotics. BMC microbiol. 2017; 17:223 10.1186/s12866-017-1133-0 29191163 PMC5709835

[107] Ahmed KS, Yasui Y, Ichikawa T. Effect of neem oil on mating and oviposition behavior of azuki bean weevil, Callosobrucus chinensis L.(Coleoptera: Bruchidae). Pak J Biol Sci. 2001; 4:1371–1373.

[108] Ryan M, Byrne O. Plant-insect coevolution and inhibition of acetylcholinesterase. J Chem Ecol. 1988; 14:1965–1975. 10.1007/BF01013489 24277106

[109] Kostyukovsky M, Rafaeli A, Gileadi C, Demchenko N, Shaaya E. Activation of octopaminergic receptors by essential oil constituents isolated from aromatic plants: possible mode of action against insect pests. Pest Manag Sci. 2002; 58:1101–1106. 10.1002/ps.548 12449528

[110] El-Wakeil NE. Retracted Article: Botanical Pesticides and Their Mode of Action. Gesunde Pflanzen. 2013; 65:125–149.

[111] Jalali Sendi J, Ebadollahi A. Biological Activities of Essential Oils on Insects. In Recent Progress in Medicinal Plants (RPMP): Essential Oils-II. 2014; 37:129–150.

[112] WHO. Temephos in drinking-water: Use for vector control in drinking-water sources and containers. Geneva: World Health Organization 2009.

[113] WHO. WHO specifications and evaluations for public health pesticides: technical temephos. World Health Organization, Geneva, Switzerland 1999.

[114] Peiris H, Hemingway J. Temephos resistance and the associated cross-resistance spectrum in a strain of Culex quinquefasciatus Say (Diptera: Culicidae) from Peliyagoda, Sri Lanka. Bull Entomol Res. 1990; 80:49–55.

[115] Tabbabi A, Daaboub J, Laamari A, Cheikh RB, Feriani M, Boubaker C, et al Evaluation of resistance to temephos insecticide in Culex pipiens pipiens larvae collected from three districts of Tunisia. Afr Health Sci. 2019; 19:1361–1367. 10.4314/ahs.v19i1.8 31148962 PMC6531957

[116] Grisales N, Poupardin R, Gomez S, Fonseca-Gonzalez I, Ranson H, Lenhart A. Temephos resistance in Aedes aegypti in Colombia compromises dengue vector control. PLoS Negl Trop Dis. 2013;7:e2438 10.1371/journal.pntd.0002438 24069492 PMC3777894

[117] Grigoraki L, Lagnel J, Kioulos I, Kampouraki A, Morou E, Labbe P, et al Transcriptome profiling and genetic study reveal amplified carboxylesterase genes implicated in temephos resistance, in the Asian tiger mosquito Aedes albopictus. PLoS Negl Trop Dis. 2015; 9: e0003771 10.1371/journal.pntd.0003771 26000638 PMC4441504

[118] Marcombe S, Chonephetsarath S, Thammavong P, Brey PT. Alternative insecticides for larval control of the dengue vector Aedes aegypti in Lao PDR: insecticide resistance and semi-field trial study. Parasit Vectors. 2018; 11:1–8. 10.1186/s13071-017-2573-y 30509299 PMC6278129

[119] Anderasen M. Emerging resistance to temephos in *Anopheles stephensi* in the Al-Dhahira Region of Oman. World Health Organization, Geneva 2006:1–13.

[120] Soltani A, Vatandoost H, Oshaghi MA, Enayati AA, Raeisi A, Eshraghian MR, et al Baseline susceptibility of different geographical strains of Anopheles stephensi (Diptera: Culicidae) to temephos in malarious areas of Iran. J Arthropod Borne Dis. 2013; 7:56–65. 23785695 PMC3684497

